# Change in quality of life over eight years in a nationally representative sample of US adults with heart disease and type 2 diabetes:minority race and toxic stress as keysocial determinants

**DOI:** 10.1186/s12889-020-08842-y

**Published:** 2020-05-14

**Authors:** Allan K. Nkwata, Xiao Song, Ming Zhang, Amara. E. Ezeamama

**Affiliations:** 1 Department of Epidemiology and Biostatistics, University of Georgia, Athens, Georgia USA; 2 Department of Psychiatry, College of Osteopathic Medicine, Michigan State University, East Lansing, MI USA

**Keywords:** Toxic stress, Minority race, Quality of life, Older Americans, Health disparities

## Abstract

**Background:**

Toxic stress (TS), minority race and their interaction are evaluated as determinants of change in quality of life (QOL) over 8 years follow-up in a nationally representative sample of United States (US) adults (≥50 years old) with heart disease (HD) and/or type-2 diabetes (T2DM) diagnosed by 2006 as part of the Health and Retirement Study (HRS).

**Methods:**

Recent and life-course stress plus experiences of lifetime discrimination were measured every 2 years using the stressful life experiences questionnaire. QOL was assessed by participant self-rated health (SRH) and operationally defined as improved, unchanged or declined in current year versus two years prior. Repeated measures multinomial logistic regressionusing generalized estimating equations (GEEs) was implemented to estimate race-, TS and their interaction- related odds of worse SRH from2006–2014. Odds ratios (OR) and 95% confidence intervals (CIs) were calculated with adjustment for time, age, sex and socio-economic status.

**Results:**

Three thousand nine hundred four adults with HD/T2DM, mean age 71.1 ± 9.3 years old, 80.9, 14.7 and 4.4% that respectively self-identified as Caucasian, African-American and Other race, were included. Over the eight-year follow-up, the odds of worse SRH for African-American and Other race were respectively 1.46 (95% CI: 1.25–1.70) and 1.43 (95% CI, 1.10–1.86) times higher relative to Caucasians. Relative to older Americans that reported ≥2 lifetime discrimination events, the odds of poor SRH was respectively 33% (OR = 0.67, 95%CI: 0.50–0.89) and 17% (OR = 0.83, 95%CI: 0.59–1.17) lower for those that reported none vs one lifetime discrimination experience. Furthermore, the relationship of life-course stress to SRH decline over 8 years varied by race (time*stress*race, *p* = 0.1173). Specifically, increasing life-course stress predicted worse QOL among Caucasians (*p* = 0.0063) and among African-American (*p* = 0.0820) but not among Other race (*p* = 0.9943).

**Conclusion:**

Toxic stress and minority race are social determinants of deterioration in QOL among older Americans with chronic diseases (HD/T2DM). The types and prevalence of toxic stressors varied by race/ethnicity. Policy interventions to address root causes of TS while targeted at proximate drivers of TS by race/ethnicity represent a viable strategy for mitigating racial disparities in overall wellbeing and improving QOL in all aging Americans regardless of race.

## Background

The population of adults at least 65 years old is steadily growing in the United States (US) and around the world [1, 2]. As of 2015 in the US, an estimated 15% (45.1 million) Americans were at least 65 years old. By the year 2050, nearly one in four Americans (approximately 83.7 million) will be at least 65 years old [1, 3]– a demographic shift expected to result in considerable burden for public health systems, medical and social services, and familycaregiving demands [4, 5]. Chronic disease prevalence and incidence – including joint diagnoses with multiple chronic conditions, are expected to rise with cancers, diabetes and heart diseases driving health care consumption and associated costs [6]. Chronic diseases lower the quality of life (QOL) for older adults and are leading drivers of disability and death in the US and around the world [7, 8]. Although the overall life-expectancy is increasing in all racial groups, notable racial disparity in life expectancy persists among US adults [9]. Furthermore, increase in life-expectancy is not accompanied by corresponding increase in years of life lived without a major health condition or healthy life expectancy (HLE) in all racial groups [10, 11]. Hence, the US National Institutes of Aging (NIA) is encouraging scientific research on understanding modifiable determinants of reduction in HLE among older Americans [12]. Additionally, elimination of racial disparities in health remains an important public health challenge and a top priority of the Healthy People 2020 program of the U.S. Department of Healthand Human Services. Empirically informed progress towards mitigating racial disparities in the USrequires an understanding of the etiologic relevance of modifiablesocial determinantssuch as psychosocial stress [13].

Well established mechanisms and theoretical frameworks, including the Stress Process Model (SPM) [14, 15] link psychosocial stress -i.e., poverty, discrimination, racism, neglect, child abuse among other socially determined factors typically beyond the control of individuals - to adverse physical health outcomes in human populations 15, 16]. These psychosocial stressors, when prolonged and combined with limited adaptive coping at the individual level, constitute toxic stress (TS) [17]. The nature of TS may evolve over the life-course [18] and manifest as negative emotional disorders like depression and anxiety, anger or hostility and external stressors resulting from repeated exposure to stressful conditions [19]. A hallmark manifestation of TS is induction of abnormal physiological responses leading to the body’s failure to fully recover from physiologic and psychosocial adversities [20]. An understanding of the contribution of TS to accelerated QOL decline in older Americans with recently diagnosed chronic disease remains unknown. This research includes adults with type 2 diabetes mellitus (T2DM) and heart disease (HD) because these chronic conditions are among the leading causes of mortality among older adultsin the US [21]. These middle age and older Americans with metabolic chronic disease represent a sensitive sub-group to investigate the health impact of psychosocial stress.

This research was further grounded in the stress process framework. Briefly, the SPM outlines three hypotheses: (a) social context shapes exposure to stressors and access to available coping resources, (b) stressors in turn negatively affect health, and (c) social and personal resources positively influence health, both directly and indirectly, by reducing the negative effects of toxic stress experiences in populations [22]. The extent to which SPM model adequately captures the psychosocial mechanisms underlying health among different racial groups in particular remains unclear because investigations in multi-ethnic US samples have been rare. A common limitation of this literature has been the implicit, tenuous assumption that relationships between psychosocial factors and health are similar across social groups [23, 24]. Such an assumption does not take into account the drastic differences in experiences and social realities faced by those located at varying intersections of race and gender hierarchies [25]. To address this limitation, we examined psychosocial stress in a multi-ethnic sample and conducted additional analyses within strata of racial groups to understand possible variations in toxic stress and relationship with wellbeing.

This longitudinal analysis used nationally representative US data from the Health and Retirement Study (HRS) during 2006 to 2014 and was designed to assess the relationship of psychosocial determinants measured at baseline (various domains of TS) to worsening SRH over 8 years amongthe insured older (i.e. 50+ years) US adults with recent diagnoses of either Type 2 Diabetes Mellitus (T2DM) or Heart Disease (HD). We hypothesized that there would be racial differences in QOL declines and that individuals experiencing higher levels of TS will have lower QOL over 8 years. Hence variations in deterioration of wellbeing during follow-up were examined with race and toxic stress as primary determinants. Specific additional analyses investigated interactions between race and TS in relationship to QOL change.

## Methods

### Study population

This was a prospective cohort study from a nationally representative sample of older Americans followed as part of the Health and Retirement Study (HRS) from 2006 to 2014. Details on the HRS design and implementation have been extensively described elsewhere [26, 27]. For the purposes of this analysis, our sample included insured adults at least 50 years old in the year 2006 who were diagnosed with T2DM and/or HD no earlier than the year 2004 and with available data on QOL and TS measures. We excluded uninsured persons, all diagnoses of HD/T2DM prior to HRS 2000, individuals < 50 years old and those lacking TS or QOL measures. Exclusion of persons without health insurance was deemed necessary, because health outcomes, TS and access to health services were likely to be fundamentally different in this sub-group. Participants were interviewed every 2 years and asked about physical health and functioning, cognitive functioning, health insurance, health care expenses, employment, and financial information such as income, assets, and pension plans [26, 27]. Our analysis period is restricted to 2006 through 2014 as psychosocial Leave-Behind questionnaires from which TS measures are determined were first administered in 2006 [28].

### Measures

#### **Primary determinants: r**ace/ethnicity and toxic stress

Race/ethnicity was self-reported and categorized as non-Hispanic Black/African American (AA), non-Hispanic White/Caucasian (White) or Other race, i.e., Hispanic or Latino [26, 27]. Toxic stress (TS) was assessed across several domains and included: recent stressors, life-course stressors, and experiences of racial discrimination [28]. Life-course stressors were determined per response to 11 questions that capture stressful life events at any point in a respondent’s life time, including loss of a child, being in a major fire, flood, earthquake, or other natural disaster, life threatening illness or major accident [28]. Recent stressors are six items that capture major stressful life events that occurred in the last 5 years namely involuntary job loss, prolonged unemployment, being robbed or burglarized, moved to a worse neighborhood, or being a victim of fraud [28]. Cumulative stress is a summation of recent stressors and life-course stressors.

Experiences of discrimination were also evaluated as a dimension of toxic stress, and these included questions asked on experiences of everyday discrimination, major experiences of lifetime discrimination and experiences of chronic work discrimination. Measures of everyday discrimination are six questions designed to tap into the hassles and chronic stress associated with perceived everyday discrimination. Major experiences of lifetime discrimination are seven questions that capture major experiences of unfair treatment at any point in one’s lifetime. Experiences of chronic work discrimination are designed to assess chronic discrimination experienced at work. These questions are only asked of respondents who are currently working and are not required for those study participants that are retired. In this set of measures, participants are asked to rate how often some stressful experiences/ situations arose at work during the last 12 months [28].

We analyzed each type of stress as a continuous variable where scores ranged from a minimum of zero to a theoretic maximum of 17 for cumulative stressors, 0–11 for life-course stress, and 0–6 for recent stress. For experiences of discrimination, the theoretic score ranged from 0 to 6 for measures of everyday discrimination, 0–7 for major experiences of lifetime discrimination, and 0–6 for experiences of chronic work discrimination.

We also analyzed each type of stress as categories based on the distribution of stress events. Cumulative stress categories and life-course stress categories included zero events (reference), one, two, and three or more events. Recent stress categories included zero events (reference), one, and two or more events. Similarly, measures of everyday, chronic work, and lifetime discrimination each included categories for zero events (reference), one, and two or more events.

#### Outcome measure: quality of life

QOL was defined per self-rated health (SRH) and self-reported in response to the question, “How would you rate your current health? Five Likert scale response options ranged from: excellent (highest),very good, good, fair, to poor (lowest). For analytic purposes, three ordinal QOL levels were defined as:poor (poor/fair), good, or excellent (i.e. very good or excellent) SRH.

### Covariates

Age was assessed by self-reported date of birth and modeled categorically in 5-year increments. Other covariates were biological sex, self-reported years of education completed, marital status and behavioral risk factors such as BMI, smoking status and current alcohol use.

### Data analysis

We implemented descriptive analyses to estimate means (with standard deviation) for continuous variables in the total sample and by race/ethnicity. For categorical variables, *T* – and *X*^2^ tests were used to examine differences by race/ ethnicity. We estimated percentages of each response of the stress measures in the total sample and by race/ ethnicity. Bivariate analyses were additionally performed to determine crude associations for each outcome with the predictors, potential confounders and socio-demographic factors. Bivariate associations were used to determine racial differences in the characteristics assessed. Factors with a *p*-value < 0.2 were further evaluated in multivariable models as candidate confounders.

To determine race-related and psychosocial status-related differences (and 95% confidence intervals) in SRH declines, we implemented repeated measures analyses for multinomial responses using generalized estimating equations (GEEs) controlling for age, sex, social economic status, marital status, BMI, smoking status and alcohol use at baseline. To accommodate correlation between repeated measures within respondents, we assumed an independent working correlation structure and modeled the odds of declining SRH (poor to good to excellent). Thus, the odds of QOL decline were determined in relation to baseline psychosocial predictors. Time was included as a class variable with values ranging from1, 2, 3 and 4 representing study years 2008, 2010, 2012, and 2014 respectively. A set of unadjusted regression models were built to quantify independent effects of race (regression model included race, time, and their interaction) and TS (regression models included TS, time, and their interaction) on four measures of QOL per respondent between 2008 and 2014. In addition to TS, race and their interactions with one another, the baseline levels of the following confounders, considering the literature and bivariate analyses, were adjusted for in multivariable models: age,sex, education and marital status. Other extraneous factors adjusted for in the models included BMI, cigarette use and alcohol consumption. Odds ratios (ORs) and 95% confidence intervals (CIs) calculated from multivariable models at alpha = 0.05.*P*-values for interaction effects were set at *p* <  0.10 because the power of statistical tests for higher order terms is generally lower than for first-order terms [29, 30].

All analyses were performed with SAS software, version 9.4 (SAS Institute, Cary, NC).

## Results

### Baseline characteristics

**Fig. 1:**
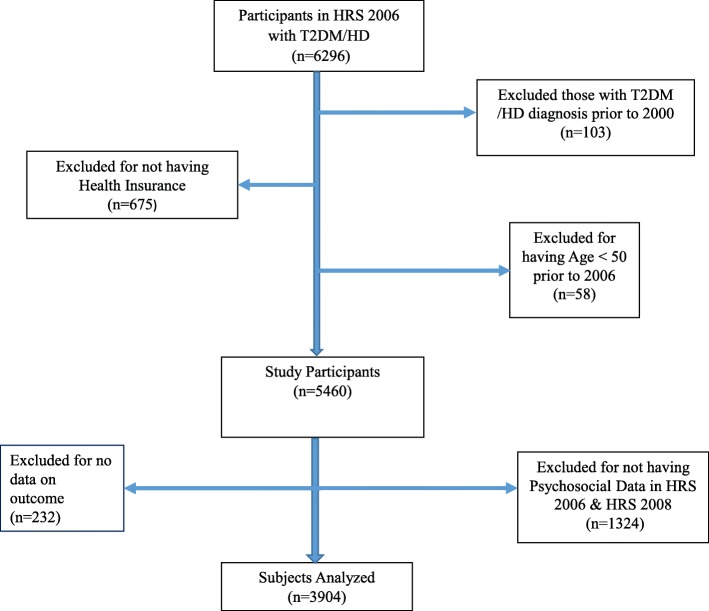
Selection of study participants from HRS 2006

A total of 6296 individuals with HD, T2DM or both conditions were identified for inclusion in the study at baseline. The baseline combined two waves of data (HRS 2006 and HRS 2008) because psychosocial questionnaires were randomly administered to half the sample in each wave. For instance, a participant who received the Psychosocial leave behind (PLB) questionnaire in 2006, had a second administration of the questionnaire in 2010, while a participant who received it in 2008, was scheduled again in 2012. Of these 103 (1.6%) were excluded with a diabetes diagnosis prior to the year 2000, 58 (0.9%) were younger than 50 years old, 675 (10.7%) lacked health insurance information, 1324 (21%) lacked stress data at baselineand 232 (3.6%) had no data on outcome measures. 3904 (65.7%) unique individuals with recent T2DM, HD or both chronic conditions were identified for analysis in the study (Fig. 1).

Their baseline demographic characteristicsby race are listed in Table 1. In brief, 3159 (80.9%) participants were Caucasian, 574 (14.7%) were African American, and 171 (04.4%) were classified as Other race. Overall, the mean (SD) age of the participants was 70.9 (±9.3) years old, 2009 (51%) were females, 2469 (63%) were married, 1934 (49.6%) were diabetic, 2525 (64.7%) had a diagnosis of heart disease (HD). However, Caucasians were on average 3 and 5 years older than African Americans and Other race:71.6 (±9.1) vs 68.6 (±8.7) and 66.6 (±9.2) years, respectively.

**Table 1:** Socio-demographic characteristics of study participants, by race at baseline

Characteristic	All Races (***N*** = 3904)	White (***N*** = 3159)	Black (A/A) (***N*** = 574)	Other (***N*** = 171)	***p***-value
	N (%)	N (%)	N (%)	N (%)	
**Age mean (SD)**	70.9 (9.3)	71.6 (9.1)	68.6 (8.7)	66.5 (9.2)	< 0.0001
**Female sex**	2009 (51.5)	1548 (49.0)	373 (65.0)	88 (51.5)	< 0.0001
**Marital Status**					
Married/ partnered	2469 (63.2)	2086 (66.0)	278 (48.4)	105 (61.4)	< 0.0001
Separated/Divorced	442 (11.3)	312 (09.9)	109 (19.0)	21 (12.3)	
Widowed	904 (23.2)	701 (22.2)	164 (28.6)	39 (22.8)	
Never married	89 (02.3)	60 (01.9)	23 (04.0)	06 (03.5)	
**Education**					
Less than High School/GED	1150 (29.5)	831 (26.3)	243 (42.3)	76 (44.4)	< 0.0001
High-school graduate	1247 (31.9)	1052 (33.3)	166 (28.9)	29 (17.0)	
Some college and above	1507 (38.6)	1276 (40.4)	165 (27.8)	66 (38.6)	
**Disease conditions**					
Diabetes	1934 (49.6)	1440 (45.6)	387 (67.4)	107 (62.6)	< 0.0001
Heart Disease	2525 (64.7)	2155 (68.2)	286 (49.8)	84 (49.1)	< 0.0001
Diabetes & Heart Disease	649 (16.9)	516 (16.6)	108 (19.2)	25 (15.4)	0.001
**Behavioral factors**					
Ever smoked	2363 (60.9)	1925 (61.3)	341 (60.0)	97 (57.4)	0.5366
Current alcohol use	1773 (45.4)	1540 (48.8)	175 (30.5)	58 (33.9)	< 0.0001
**Life-course stressors**					
0 events	734 (19.2)	567 (18.3)	129 (23.3)	38 (23.0)	0.0524
1 event	1041 (27.2)	857 (27.6)	144 (26.0)	40 (24.2)	
2 events	888 (23.2)	737 (23.8)	121 (21.8)	30 (18.2)	
3+ events	1157 (30.3)	940 (30.3)	160 (28.9)	57 (34.6)	
**Recent stressors**					
0 events	3209 (82.2)	2641 (83.6)	443 (77.2)	125 (73.1)	< 0. 0001
1 event	533 (13.7)	398 (12.6)	105 (18.3)	30 (17.5)	
2+ events	162 (04.1)	120 (03.8)	26 (04.5)	16 (09.4)	
**Cumulative stressors**					
0 events	662 (17.3)	518 (16.7)	111 (20.0)	33 (20.0)	0.2315
1 event	937 (24.5)	769 (24.8)	133 (24.0)	35 (21.2)	
2 events	878 (23.0)	731 (23.6)	115 (20.8)	32 (19.4)	
3+ events	1343 (35.1)	1083 (34.9)	195 (35.2)	65 (39.4)	
**Lifetime Discrimination**					
0 events	2694 (70.0)	2236 (71.6)	348 (62.0)	110 (65.1)	< 0.0001
1 event	689 (17.9)	559 (17.9)	94 (16.8)	36 (21.3)	
2+ events	468 (12.1)	326 (10.5)	119 (21.2)	23 (13.6)	
**Everyday Discrimination**					
0 events	1742 (45.0)	1429 (45.6)	238 (41.9)	75 (44.4)	0.2252
1 event	1133 (29.3)	914 (29.2)	164 (28.9)	55 (32.5)	
2+ events	994 (25.7)	789 (25.2)	166 (29.2)	39 (23.1)	
**Chronic work Discrimination**					
0 events	764 (84.9)	600 (85.7)	119 (81.5)	45 (83.3)	0.4782
1+ events	136 (15.1)	100 (14.3)	27 (18.5)	09 (16.7)	
**Self-rated health**					
Excellent	865 (22.2)	758 (24.0)	81 (14.1)	26 (15.2)	< 0.0001
Good	1384 (35.4)	1161 (36.8)	171 (29.8)	52 (30.4)	
Poor	1653 (42.4)	1238 (39.2)	322 (56.1)	93 (54.4)	

Overall median recent stressors were 0 (IQR:0,0), life-course stressors 2 (IQR: 1.0, 3.0), cumulative stressors 2.0 (IQR: 1.0, 3.0), experiences of daily discrimination 1 (IQR: 0,1) major experiences of lifetime discrimination 0 (IQR: 0, 1) and experiences of chronic work discrimination 0 (IQR: 0,0).

Additional baseline characteristics are reported in Supplementary Tables 1, 2, 3, 4, and 5 showing the distributions of toxic stress questions at baseline by race for each domain of toxic stress assessed.

### Association of race on change in QOL

Race-ethnicity was an independent, strong and time-invariant determinant of change in SRH in this sample afteradjusting for the following covariates at baseline; education, sex, age, BMI, smoking status, alcohol use and marital status. Specifically, African Americans and Other race had 46% (OR 1.46, 95% CI: 1.25, 1.70) and 43% (OR 1.43, 95% CI: 1.10, 1.86) higher odds ofpoorer SRH respectively relative to older Caucasian Americans. The association between race/ethnicity and patient reported SRH change was stable over 8 years of follow-up (time*race, *p* = 0.6575), thus time-averaged associations are provided. SRH change over time was similar inAfrican Americans and Other race (Table 2).

**Table 2:** Time-averaged effects of baseline toxic stressors on SRH-declines reported by HRS study participants over the eight-year follow-up

		Odds Ratio (95% CI)	***p***-Value Race	***p***-Value T*Race
**Race**	Black/AA vs White/Caucasian	**1.46 (1.25, 1.70)**	< 0.0001	0.6575
	Other vs White/Caucasian	**1.43 (1.10, 1.86)**		
	Black/AA vs Other	1.02 (0.76, 1.37)		
**Toxic stressor types**	**Intensity of stressor**		**p-Value Stress**	p-Value T*Stress
**Everyday Discrimination**	0 vs 2+ Day discrimination	0.92 (0.71, 1.18)	0.8484	0.1960
	1 vs 2+ Day discrimination	0.95 (0.73, 1.25)		
**Lifetime Discrimination**	0 vs 2+ Lifetime discrimination	**0.67 (0.50, 0.89)**	0.0111	0.0950
	1 vs 2+ Lifetime discrimination	0.83 (0.59, 1.17)		
**Chronic work discrimination**	0 vs 1+ Work discrimination	0.95 (0.51, 1.78)	0.8948	0.4059

Notes: *CI* confidence interval. Odds ratios in bold are statistically significant. Models adjusted for race, age, sex, education, marital status, BMI, cigarette use, alcohol consumption and interaction terms for race*time, stress*time as well as three -way terms for race*stress*time

### Association of toxic stress domains on SRH declines

Major experiences of lifetime discrimination were strongly associated with higher odds for SRH change over time (*p* = 0.095). Regardless of time interval, having none vs two or more major experiences of lifetime discrimination was associated with 33% lower odds of poorer SRH (OR 0.67, 95% CI: 0.50, 0.89). Similarly, having one vs two or more major experiences of lifetime discrimination was associated with 17% lower odds of SRH change, although this was not statistically significant (OR 0.83, 95% CI: 0.60, 1.17). A dose-dependent decrease in likelihood of poorer SRH was observed. Experiences of everyday and chronic work discrimination were not associated with poorer SRH (Table 2).

Recent stress-related changes in SRH over 8 years follow-up varied by race (race*time*stress, *p* = 0.0809). Among older Caucasians, the association between recent stress and poorer SRH did not vary over time (time*stress, *p* = 0.1286) and there was no significant association between experiences of none or one vs. two recent stressors in any study interval. Among older African Americans, however, the association between having none or one vs two or more recent stressors strengthened over time (and marginally significant in 2012) to become more protective for SRH declines (time*stress, *p* = 0.033). Among older Americans of Other race, there was no significant association between having none or one vs two or more recent stressors on SRH declines over time (time*stress, *p* = 0.5895) (Table 3).

**Table 3:** Toxic stressors as a determinant of Decline in Self-Rated Health (QOL) over 8 years in the Overall Sample and Within Race/Ethnicity Stratum

	2008	2010	2012	2014	Stress	Time*Stress	Race*Time*Stress
**Toxic Stress Domain**	**Odds Ratio (95% CI)**	**Odds Ratio (95% CI)**	**Odds Ratio (95% CI)**	**Odds Ratio (95% CI)**	**P-value**	**P-value**	**P-value**
**Recent Stressors**							
**Entire Sample Regardless of Race**							
0 vs 2+ Recent stressors	1.04 (0.68, 1.60)	1.08 (0.67, 1.74)	0.74 (0.44, 1.26)	0.68 (0.37, 1.23)	0.7799	0.359	**0.0809**
1 vs 2+ Recent stressors	1.14 (0.70, 1.88)	0.99 (0.57, 1.73)	0.66 (0.37, 1.18)	0.73 (0.37, 1.42)			
**Among Older White Americans**							
0 vs 2+ Recent stressors	0.76 (0.53, 1.09)	0.94 (0.65, 1.35)	1.03 (0.70, 1.53)	0.95 (0.63, 1.43)	0.5461	0.1286	n/a
1 vs 2+ Recent stressors	0.97 (0.66, 1.43)	0.99 (0.66, 1.49)	1.01 (0.65, 1.56)	1.07 (0.67, 1.69)			
**Among Older African Americans**							
0 vs 2+ Recent stressors	1.11 (0.51, 2.42)	0.87 (0.36, 2.08)	**0.38 (0.15, 0.99)**	0.42 (0.14, 1.23)	0.4727	**0.033**	n/a
1 vs 2+ Recent stressors	0.77 (0.34, 1.79)	0.79 (0.31, 2.02)	0.40 (0.15, 1.10)	0.56 (0.18, 1.77)			
**Among Older Other race**							
0 vs 2+ Recent stressors	1.34 (0.51, 3.51)	1.55 (0.52, 4.57)	1.02 (0.30, 3.49)	0.81 (0.21, 3.16)	0.6733	0.5895	n/a
1 vs 2+ Recent stressors	2.00 (062, 6.43)	1.26 (0.34, 4.65)	0.70 (0.18, 2.82)	0.64 (0.13, 3.11)			
**Life-course stressors**							
**Entire Sample Regardless of Race**							
0 vs 3+ life-course stressors	0.81 (0.59, 1.12)	0.79 (0.55 1.12)	0.77 (0.53, 1.12)	**0.62 (0.42, 0.92)**	0.1863	**0.1227**	**0.1173**
1 vs 3+ life-course stressors	**0.67 (0.49, 0.92)**	0.97 (0.69, 1.35)	0.94 (0.67, 1.32)	**0.69 (0.48, 0.99)**			
2 vs 3+ life-course stressors	0.87 (0.62, 1.22)	0.95 (0.67, 1.36)	0.84 (0.57, 1.19)	**0.63 (0.42, 0.95)**			
**Among Older White Americans**							
0 vs 3+ life-course stressors	**0.76 (0.62, 0.92)**	**0.78 (0.64, 0.97)**	**0.73 (0.58, 0.91)**	0.86 (0.68, 1.10)	**0.0063**	0.6654	n/a
1 vs 3+ life-course stressors	**0.72 (0.61, 0.87)**	**0.79 (0.66, 0.96)**	**0.71 (0.58, 0.87)**	0.85 (0.68, 1.09)			
2 vs 3+ life-course stressors							
	**0.80 (0.66, 0.96)**	0.92 (0.76, 1.12)	0.86 (0.69, 1.07)	0.98 (0.78, 1.24)			
**Among Older African Americans**							
0 vs 3+ life-course stressors	0.88 (0.56, 1.42)	0.69 (0.43, 1.12)	**0.48 (0.29, 0.79)**	**0.47 (0.27, 0.83)**	0.0828	0.3159	n/a
1 vs 3+ life-course stressors	0.75 (0.46, 1.20)	0.72 (0.45, 1.15)	0.73 (0.44, 1.20)	0.59 (0.33, 1.04)			
2 vs 3+ life-course stressors	0.72 (0.45, 1.14)	0.83 (0.51, 1.36)	0.80 (0.47, 1.35)	0.72 (0.41, 1.26)			
**Among Older Other race**							
0 vs 3+ life-course stressors	0.80 (0.35, 1.83)	0.90 (0.35, 2.27)	1.24 (0.46, 3.33)	0.59 (0.22, 1.61)	0.9943	0.0204	n/a
1 vs 3+ life-course stressors	0.55 (0.25, 1.23)	1.58 (0.67, 3.75)	1.58 (0.66, 3.75)	0.66 (0.27, 1.65)			
2 vs 3+ life-course stressors	1.15 (0.48, 2.77)	1.12 (0.44, 2.86)	0.83 (0.33, 2.04)	0.36 (0.12, 1.04)			
**Cumulative stressors**							
**Entire Sample Regardless of Race**							
0 vs 3+ Cumulative stressors	0.83 (0.60, 1.15)	0.76 (0.54, 1.08)	0.75 (0.51, 1.11)	0.68 (0.45, 1.01)	0.361	**0.1035**	**0.0282**
1 vs 3+ Cumulative stressors	0.77 (0.56, 1.07)	1.24 (0.89, 1.74)	1.06 (0.74, 1.51)	0.86 (0.59, 1.24)			
2 vs 3+ Cumulative stressors	0.95 (0.69, 1.32)	1.03 (0.73, 1.46)	0.85 (0.61, 1.19)	0.83 (0.56, 1.23)			
**Among Older White Americans**							
0 vs 3+ Cumulative stressors	**0.73 (0.60, 0.89)**	**0.79 (0.64, 0.98)**	**0.78 (0.62, 0.98)**	0.82 (0.65, 1.05)	**0.0091**	0.8348	n/a
1 vs 3+ Cumulative stressors	**0.74 (0.62, 0.88)**	**0.79 (0.66, 0.96)**	**0.76 (0.62, 0.92)**	0.89 (0.71, 1.11)			
2 vs 3+ Cumulative stressors	**0.83 (0.70, 0.99)**	0.94 (0.78, 1.14)	0.92 (0.74, 1.13)	0.98 (0.79, 1.23)			
**Among Older African Americans**							
0 vs 3+ Cumulative stressors	1.04 (0.65, 1.66)	0.69 (0.43, 1.11)	**0.44 (0.27, 0.74)**	**0.45 (0.26, 0.79)**	**0.0934**	**0.1424**	n/a
1 vs 3+ Cumulative	0.84 (0.53, 1.33)	0.83 (0.53, 1.31)	0.71 (0.43, 1.16)	**0.56 (0.32, 0.97)**			
stressors							
2 vs 3+ Cumulative stressors	1.04 (0.66, 1.63)	0.91 (0.56, 1.48)	0.76 (0.46, 1.26)	0.82 (0.46, 1.44)			
**Among Older Other race**							
0 vs 3+ Cumulative stressors	0.76 (0.33, 1.75)	0.82 (0.33, 2.02)	1.34 (0.54, 3.29)	0.83 (0.29, 2.33)	0.452	**0.0973**	n/a
1 vs 3+ Cumulative stressors	0.75 (0.33, 1.72)	**2.93 (1.22, 7.08)**	2.23 (0.90, 5.55)	1.27 (0.51, 3.20)			
2 vs 3+ Cumulative stressors	1.00 (0.43, 2.33)	1.28 (0.52, 3.17)	0.89 (0.38, 2.08)	0.72 (0.26, 1.98)			

Notes: *CI* confidence interval. Odds Ratios in bold are statistically significant. Models adjusted for race, age, sex, education, marital status, BMI, cigarette use, alcohol consumption and interaction terms for race*time, stress*time as well as three -way terms for race*stress*time

Life-course stress-related changes in SRH varied over time and by race (time*stress*race, *p* = 0.1173). The protective association between having none or one or two vs three or more life-course stressors and SRH declines strengthened over time becoming significant at study end across all dose-dependent categories (time *stress, *p* = 0.1227). Amongst older Caucasians, life-course stressors were associated with decreased odds of SRH changeover time (*p* = 0.0063). The protective association of having none or one or two vs three or more life-course stressors on SRH change was significant at baseline and stayed stable throughout the study period (time*stress, *p* = 0.6654). Among older African Americans, the association between life-course stressors and change in SRH did not vary over time (time*stress, *p* = 0.3159). However, the protective association of none vs three or more life-course stressors grew over time becoming significant at two study intervals during follow up. Among older Americans of Other race, the association between having none or one or two vs three or more life-course stressors strengthened over time to become more protective for SRH declinesby study end (time*stress, *p* = 0.0204) (Table 3).

Cumulative stress related declines in SRH varied over time and by race (time*stress*race, *p* = 0.0282). The protective association of having none or one or two vs three or more life-course stressors on SRH declines increased over time and was strongest at the end of study. Amongst older Caucasians, cumulative stressors were associated with decreased odds of SRH declinesover time (*p* = 0.0091). The protective association of having none or one or two vs three or more cumulative stressors on SRH declines was significant at baseline and stayed stable throughout the study period.

Among older African Americans, the association between cumulative stressors and poorer SRH did not vary over time (time*stress, *p* = 0.1424). However, the association between the experience of none or one vs three or more cumulative stressors strengthened over time becoming significant at two study intervals during follow up. Among older Americans of Other race, the association between having none or one or two vs three or more cumulative stressors varied over time (time*stress, *p* = 0.0973). Of note, having one vs three cumulative stressors was associated with 193% higher odds for SRH declines in the second time interval (OR: 2.93, 95%CI: 1.22, 7.08) (Table 3).

## Discussion

In this representative sample of vulnerable older Americans of average age 71+ years and with comorbid T2DM and/or HD at enrollment, SRH declined significantly as a function of aging and existing morbidity over the eight-year follow-up. Furthermore, we evaluated the heterogeneous impact of cumulative toxic stress by race/ ethnicity- a novel feature. In line with our hypothesis, higher TS predicted faster QOL decline in Whites and AfricanAmericans. Above and beyond the expected impact of time and comorbid disease, race and TS-dependent differences in SRH declines were evident after adjusting for education, sex, age, BMI, smoking status, alcohol use and marital status. In line with our hypothesis, increasing numbers of life-course stressors, cumulative stressors, and major experiences of lifetime discrimination were associated with poorer SRH over time. These findings were consistent with prior research which showed increasing levels of TS to be a strong predictor of incident T2DM [31]. Our study, however, further showed that among persons with HD and T2DM, higher TS levels is associated with more rapid QOL decline. Experiences of every day discrimination were marginally predictive of poorer SRH. Recent stressors, experiences of chronic work discrimination, however, were not associated with change in SRH.

We also found higher odds of QOL decline for minority races vs. Caucasian Americans. These findings corroborate those from another study that explored how acculturation characteristics, social class, marital status, and chronic illness mediate or moderate differences in non-specific psychological distress for eight racial/ethnic populations in the US. The authors found that non-White populations had variable baseline differences in psychological distress compared to Whites; however, this variation was not connected to health outcomes, a gap that our study informs [32]. Our findings also corroborate those by Sternthal and colleagues, who found a higher prevalence and greater clustering of high stress scores in African Americans than in Caucasians. They also found comparable stress scores between American-born and African Americans, and similar scoresbetween foreign-born Hispanics and Caucasians. In their study, multiple stressors were associated with poor physical and mental health outcomes [33].

With regards to the effect of stressors on health, our findings are consistent with extant literature on racial disparities in health outcomes in the US [34, 35]. Of note, literature on stress exposure has typically focused on acute life events, thus limiting the range of stressors examined [36, 37]. Furthermore, with a few exceptions, most studies on racial differences in stress exposure compare distributions of stressors among Blacks versus Whites, to the exclusion of Hispanics. This is problematic because Hispanics who are now the largest minority population, are over represented in the lower socioeconomic stratum, and experience high levels of stressors related to acculturation, job hazards, poverty, and legal residency/citizenship status [38, 39]. In addition, results from past research on race-related variations in stress domains have been variable [24]. Majority of past research that compared levels of distress between African Americans and Caucasian Americans found similar levels of psychological distress between the two groups [40–42]. The finding in this sample that overall levels of toxic stress is similar across racial groups is consistent with these prior reports [40, 41].

However, the potential heterogeneity by race in types of stressors and their adverse health effects on health within older US adults as implemented herein is novel. For example, regardless of race, high lifetime discrimination was associated with lower QOL, but participant-reported experience of any or multiple experiences of lifetime discrimination was highest among African Americans, followed by Other race and lowest in Caucasian Americans. This finding is consistent with prior reports linking the experience of discrimination with negative health effects and poor work-place productivity [43–46]. Relative to Caucasian Americans, individuals of minority race are more likely to live in neighborhoods with concentrated disadvantages – a factor that partly explains the higher prevalence of stress exposure among African Americans and Hispanics relative to Caucasians [47]. Due to segregation, the conditions under which African Americans and Hispanics live are far worse than those of the rest of the population. For those residing in areas of concentrated disadvantage, with poor physical and social conditions such as extreme poverty and unemployment, pollution, deteriorating housing and violence, multiple stressful encounters may be the norm [33, 37]. Also, minorities have for a long time been socially and economically deprived, exposed to toxic substances and hazardous working conditions, experienced more physical and mental trauma, unequal law enforcement and protection via the court system, lived in areas with food deserts, and limited access to adequate medical care among other things [48]. Addressing these and other extenuating factors would improve QOL among African Americans. Data from our study suggests benefits of policy interventions that reduce TS psychosocial stress among African Americans could result in long-lasting progressively protective effects over time. Of note, we specifically evaluated whether our study results were consistent with the intersectionality theory of race/stress effects on QOL – i.e. that inequities in QOL by race were mediated by experiences through discrimination and psychosocial stress [49]. This theory was not confirmed as our results show that minority race/ethnicity was a stable time-invariant predictor of worse QOL over 8 years of follow-up. Higher levels of psychosocial stress were an independent determinant of wellbeing regardless of race. However, the salient indicator of psychosocial stress in relationship to change in QOL varied across racial groups (Supplementary Table 6).

Although subjective self-reported exposure (toxic stressors) and outcome (SRH) measures are the gold standard for these assessments, patient-reported measures are inherently variable and thus a limitation of this research. Specific elements of design including collection of these data using standardized and validated questionnaires, with known psychometric properties assure that these subjective measures are consistently quantified in the study base. Further, toxic stress scores have been defined as the sum of equally weighted individual questions which assumes that all stressful events have the same weight for the individual. This assumption may over-simplify more complicated phenomena; however, our empirical goal was to analyze cumulative stress within the ACES (adverse childhood experiences) framework and relate these to health outcomes. Because stress perception is self -reported and ultimately subjective relative to the individual, the negative valence of any one factor is unlikely to be the same across our entire sample. Hence, we considered equal weighting of factors to be a reasonable strategy for quantifying cumulative stress and this is approach has precedence in previous reports by our research group and others [31, 50, 51].

This study features key strengths that should increase confidence in the reported findings. Specifically, we implemented a large prospective cohort study of older Americans with metabolic chronic disease where each participant had four repeated measures over 8 years of follow-up. Hence, our design permitted evaluation of change in QOL in relation to toxic stress measures and we employed rigorous analytic techniques that controlled for several potential confounding factors. Lastly, we studied toxic stressors holistically by evaluating them across several dimensions; recent, life-course stressors and experiences of racial discrimination and thus substantially contribute to an understanding of the potentially modifiable role of various forms of toxic stress on change in QOL in a diverse sample of older Americans.

## Conclusion

The present study evaluated TS and minority race as determinants of quality of life (QOL) decline in a nationally representative sample of≥50 years old United States (US) adults with heart disease (HD) and/or type-2 diabetes (T2DM). Among older Americans with HD and T2DM, minority race and higher TS levels are social determinants of decline in wellbeing. Our findings are important given that we live in a race-conscious society in which racism still abounds on multiple levels, including institutionalized, personally mediated, and internalized, each of which can have negative impacts on health [52]. Our results provide empirical evidence that social, economic and health policies that address structural inequities in social experiences that shape exposure to a broad range of environmental stressors are likely to translate to improved wellbeing in a broad section of older US adults [33]. For example, social policies may be targeted accordingly to reduce community level TS known to vary along racial lines in the US such as: experiences with law enforcement with expected onward benefit for reducing race-related disparities in wellbeing observed in this representative sample of US adults.

## Supplementary information

**Additional file 1.**

## Data Availability

The data that support the findings of this study are publicly available as HRS public release data files which can be accessed at https://hrs.isr.umich.edu/data-products/access-to-public-data.

## Abbreviations

TS: Toxic Stress
QOL: Quality of Life
US: United States of America
HD: Heart Diseases
T2DM: Type 2 Diabetes
HRS: Health and Retirement Study
AA: African American
NIA: National Institutes of Aging
SSA: Social Security Administration
HLE: Healthy Life Expectancy
SRH: Self-rated Health
SPM: Stress Process Model
