# Correction to: Change in quality of life over eight years in a nationally representative sample of US adults with heart disease and type 2 diabetes: minority race and toxic stress as key social determinants

**DOI:** 10.1186/s12889-020-09399-6

**Published:** 2020-09-01

**Authors:** Allan K. Nkwata, Xiao Song, Ming Zhang, Amara. E. Ezeamama

**Affiliations:** 1 Department of Epidemiology and Biostatistics, University of Georgia, Athens, Georgia USA; 2 Department of Psychiatry, College of Osteopathic Medicine, Michigan State University, East Lansing, MI USA

**Correction to: BMC Public Health 20, 684 (2020)**

**https://doi.org/10.1186/s12889-020-08842-y**

This Correction article is to inform readers of the following errors in the original publication of this article [1]:

1. Dr. Ezeamama is also a corresponding author of the article. Dr. Ezeamama helped conceive the ideas underlying this published study and has closely overseen and guided every progress of the project as the first author’s major advisor for the past four years.
  The correspondence details are included in this correction article.
2. The title should read “key social”
