# Supplementary material for: Change in quality of life over eight years in a nationally representative sample of US adults with heart disease and type 2 diabetes:minority race and toxic stress as keysocial determinants
Source: BMC Public Health. 2020 May 14;20:684. doi: 10.1186/s12889-020-08842-y (PMC7222334; doi:10.1186/s12889-020-08842-y)
Supplement: Supplementary file 1 — Additional file 1. [file 12889_2020_8842_MOESM1_ESM.docx]

## Supplementary Tables- Toxic Stress Manuscript

The following tables show the distribution of our toxic stress questions at baseline by race. Questions are in their raw form before scores that are used in the analyses are derived. Scores were derived per guidelines in the HRS Psychosocial Leave Behind questionnaire (<https://hrs.isr.umich.edu/sites/default/files/biblio/HRS%202006-2016%20SAQ%20Documentation_07.06.17.pdf>).

| **Supplementary Table 1: Distribution of Day to day discrimination items at baseline by race** | | | | | |
| --- | --- | --- | --- | --- | --- |
|  | **All Races (N=3904)** | **White (N=3159)** | **Black (A/A) (N=574)** | **Other (N=171)** |  |
| **Experiences of Day to day discrimination** | **N (%)** | **N (%)** | **N (%)** | **N (%)** | **p-value** |
| 1. You are treated with less courtesy and respect than other people. |  |  |  |  | < 0.0001 |
| Never | 1747 (45.4) | 1404 (45.0) | 261 (43.4) | 82 (48.8) |  |
| Less than once a year | 790 (20.5) | 689 (22.1) | 72 (12.8) | 29 (17.3) |  |
| A few times a year | 782 (20.3) | 644 (20.7) | 113 (20.1) | 25 (14.9) |  |
| A few times a month | 281 (07.3) | 211 (06.8) | 55 (09.8) | 15 (08.9) |  |
| At least once a week | 144 (03.7) | 99 (03.2) | 37 (06.6) | 08 (04.8) |  |
| Almost every day | 103 (02.7) | 69 (02.2) | 25 (04.4) | 09 (05.4) |  |
| 2. You receive poorer service than other people at restaurants or stores. | |  |  |  | < 0.0001 |
| Never | 2297 (59.7) | 1871 (60.0) | 323 (57.5) | 103 (62.0) |  |
| Less than once a year | 826 (21.5) | 700 (22.4) | 93 (16.6) | 33 (19.9) |  |
| A few times a year | 500 (13.0) | 394 (12.6) | 90 (16.0) | 16 (09.6) |  |
| A few times a month | 145 (03.8) | 101 (03.2) | 36 (06.4) | 08 (04.8) |  |
| At least once a week | 54 (01.4) | 38 (01.2) | 15 (02.7) | 01 (0.6) |  |
| Almost every day | 26 (0.7) | 16 (0.5) | 05 (0.9) | 05 (03.0) |  |
| 3. People act as if they think you are not smart. |  |  |  |  | < 0.0001 |
| Never | 2216 (57.7) | 1800 (57.9) | 315 (56.0) | 101 (59.8) |  |
| Less than once a year | 694 (18.1) | 603 (19.4) | 69 (12.3) | 22 (13.0) |  |
| A few times a year | 552 (14.4) | 437 (14.1) | 94 (16.7) | 21 (12.4) |  |
| A few times a month | 195 (05.1) | 143 (04.6) | 41 (07.3) | 11 (06.5) |  |
| At least once a week | 102 (02.7) | 74 (02.4) | 22 (03.9) | 06 (03.6) |  |
| Almost every day | 82 (02.1) | 52 (01.7) | 22 (03.9) | 08 (04.7) |  |
| 4. People act as if they are afraid of you. |  |  |  |  | 0.0049 |
| Never | 3049 (79.1) | 2476 (79.4) | 444 (78.9) | 129 (76.8) |  |
| Less than once a year | 387 (10.0) | 328 (10.5) | 43 (07.6) | 16 (09.5) |  |
| A few times a year | 248 (06.4) | 200 (06.4) | 37 (06.6) | 11 (06.5) |  |
| A few times a month | 91 (02.4) | 66 (02.1) | 19 (03.4) | 06 (03.6) |  |
| At least once a week | 46 (01.2) | 33 (01.0) | 11 (02.0) | 02 (01.2) |  |
| Almost every day | 29 (0.7) | 16 (0.5) | 09 (01.6) | 04 (02.4) |  |
| 5. You are threatened or harassed. |  |  |  |  | 0.0047 |
| Never | 3161 (82.1) | 2554 (81.9) | 466 (82.9) | 141 (83.9) |  |
| Less than once a year | 423 (11.0) | 366 (11.7) | 49 (08.7) | 08 (04.8) |  |
| A few times a year | 171 (04.4) | 131 (04.2) | 28 (05.0) | 12 (07.1) |  |
| A few times a month | 49 (01.3) | 34 (01.1) | 09 (01.6) | 06 (03.6) |  |
| At least once a week | 28 (0.7) | 20 (0.6) | 07 (01.2) | 01 (0.6) |  |
| Almost every day | 17 (0.4) | 14 (0.4) | 03 (0.5) | 0 (0) |  |
| 6. You receive poorer service or treatment than other people from doctors or hospitals. * |  |  |  |  | 0.0012 |
| Never | 1516 (80.1) | 1238 (80.5) | 209 (78.3) | 69 (79.3) |  |
| Less than once a year | 219 (11.6) | 183 (11.9) | 29 (10.9) | 07 (08.0) |  |
| A few times a year | 107 (05.7) | 88 (05.7) | 14 (05.2) | 05 (05.8) |  |
| A few times a month | 25 (01.3) | 11 (0.7) | 10 (03.8) | 04 (04.6) |  |
| At least once a week | 14 (0.7) | 09 (0.6) | 03 (1.1) | 02 (02.3) |  |
| Almost every day | 11 (0.6) | 09 (0.6) | 02 (0.7) | 0 (0) |  |
| Note: Baseline stress measures combined the HRS waves 2006 and 2008 since the PLB questionnaires were randomly administered to half the sample in each wave. *=Question asked beginning in HRS 2008; score was rescaled for HRS 2006 to make it easy to compare data between 2006 and 2008. | | | | | |

| **Supplementary Table 2: Distribution of Life time discrimination items at baseline by race** | | | | | |
| --- | --- | --- | --- | --- | --- |
|  | **All Races (N=3904)** | **White (N=3159)** | **Black (A/A) (N=574)** | **Other (N=171)** |  |
| **Experiences of Life time discrimination** | **N (%)** | **N (%)** | **N (%)** | **N (%)** | **p-value** |
| 1.  At any time in your life, have you ever been unfairly dismissed from a job? |  |  |  |  | 0.8508 |
| No | 3214 (83.5) | 2600 (83.4) | 471 (84.3) | 143 (84.1) |  |
| Yes | 634 (16.5) | 519 (16.6) | 88 (15.7) | 27 (15.9) |  |
| 2.  For unfair reasons, have you been hired for a job? |  |  |  |  | 0.0003 |
| No | 3494 (91.3) | 2863 (92.1) | 480 (87.0) | 151 (90.4) |  |
| Yes | 332 (08.7) | 244 (07.9) | 72 (13.0) | 16 (09.6) |  |
| 3.  Have you ever been unfairly denied a promotion? |  |  |  |  | < 0.0001 |
| No | 3388 (88.9) | 2790 (90.1) | 454 (82.7) | 144 (86.2) |  |
| Yes | 423 (11.1) | 305 (09.9) | 95 (17.3) | 23 (13.8) |  |
| 4. Have you ever been unfairly prevented from moving into a neighborhood because the landlord or a realtor refused to sell or rent you a house or apartment? |  |  |  |  | < 0.0001 |
| No | 3742 (97.1) | 3076 (98.4) | 508 (91.2) | 158 (93.5) |  |
| Yes | 110 (02.9) | 50 (01.6) | 49 (08.8) | 11 (06.5) |  |
| 5.  Have you ever been unfairly denied a bank loan? |  |  |  |  | < 0.0001 |
| No | 3633 (94.5) | 2995 (96.0) | 481 (86.7) | 157 (92.9) |  |
| Yes | 210 (05.5) | 124 (04.0) | 74 (13.3) | 12 (07.1) |  |
| 6.  Have you ever been unfairly stopped, searched, questioned, physically threatened or abused by the police? |  |  |  |  | < 0.0001 |
| No | 3655 (94.8) | 3015 (96.4) | 488 (87.6) | 152 (89.4) |  |
| Yes | 199 (05.2) | 112 (03.6) | 69 (12.4) | 18 (10.6) |  |
| 7.  Have you ever been unfairly denied health care or treatment? * |  |  |  |  | 0.0003 |
| No | 1821 (96.8) | 1494 (97.3) | 241 (92.7) | 86 (98.9) |  |
| Yes | 61 (03.2) | 41 (02.7) | 19 (07.3) | 01 (01.1) |  |
| Note: Baseline stress measures combined the HRS waves 2006 and 2008 since the PLB questionnaires were randomly administered to half the sample in each wave. *= Question asked beginning in HRS 2008; score was rescaled for HRS 2006 to make it easy to compare data between 2006 and 2008. | | | | | |

| **Supplementary Table 3: Distribution of Chronic work discrimination items at baseline by race** | | | | | |
| --- | --- | --- | --- | --- | --- |
|  | **All Races (N=3904)** | **White (N=3159)** | **Black (A/A) (N=574)** | **Other (N=171)** |  |
| **Experiences of Chronic work discrimination** | **N (%)** | **N (%)** | **N (%)** | **N (%)** | **p-value** |
| 1. How often are you unfairly given the tasks at work that no one else wants to do? |  |  |  |  | 0.4457 |
| Never | 499 (55.9) | 382 (55.00) | 86 (59.7) | 31 (58.5) |  |
| Less than once a year | 116 (13.0) | 97 (14.0) | 14 (09.7) | 05 (09.5) |  |
| A few times a year | 137 (15.4) | 109 (15.7) | 22 (15.3) | 06 (11.3) |  |
| A few times a month | 66 (07.4) | 51 (07.3) | 10 (06.9) | 05 (09.4) |  |
| At least once a week | 48 (05.4) | 36 (05.2) | 06 (04.2) | 06 (11.3) |  |
| Almost every day | 26 (02.9) | 20 (02.9) | 06 (04.2) | 00 (0) |  |
| 2. How often are you watched more closely than others? |  |  |  |  | 0.3088 |
| Never | 615 (69.3) | 489 (70.7) | 91 (64.1) | 35 (64.8) |  |
| Less than once a year | 102 (11.5) | 84 (12.1) | 13 (09.1) | 05 (09.3) |  |
| A few times a year | 65 (07.3) | 47 (06.8) | 13 (09.1) | 05 (09.3) |  |
| A few times a month | 42 (04.7) | 31 (04.5) | 09 (06.3) | 02 (03.7) |  |
| At least once a week | 25 (02.8) | 16 (02.3) | 06 (04.2) | 03 (05.6) |  |
| Almost every day | 39 (04.4) | 25 (03.6) | 10 (07.0) | 04 (07.4) |  |
| 3. How often are you bothered by your supervisor or co-workers making slurs or jokes about women or racial or ethnic groups? |  |  |  |  | 0.2077 |
| Never | 696 (78.9) | 550 (79.8) | 108 (77.1) | 38 (71.7) |  |
| Less than once a year | 76 (08.6) | 59 (08.6) | 12 (08.6) | 05 (09.4) |  |
| A few times a year | 54 (06.1) | 41 (06.0) | 07 (05.0) | 06 (11.3) |  |
| A few times a month | 26 (03.0) | 21 (03.0) | 04 (02.9) | 01 (01.9) |  |
| At least once a week | 18 (02.0) | 13 (01.9) | 04 (02.9) | 01 (01.9) |  |
| Almost every day | 12 (01.4) | 05 (0.7) | 05 (03.6) | 02 (03.8) |  |
| 4. How often do you feel that you have to work twice as hard as others at work? |  |  |  |  | 0.3442 |
| Never | 564 (63.2) | 447 (64.3) | 86 (60.1) | 31 (57.4) |  |
| Less than once a year | 48 (05.4) | 36 (05.2) | 07 (04.9) | 05 (09.3) |  |
| A few times a year | 104 (11.7) | 83 (11.9) | 18 (12.6) | 03 (05.6) |  |
| A few times a month | 59 (06.6) | 46 (06.6) | 08 (05.6) | 05 (09.3) |  |
| At least once a week | 50 (05.6) | 36 (05.2) | 08 (05.6) | 06 (11.1) |  |
| Almost every day | 67 (07.5) | 47 (06.8) | 16 (11.2) | 04 (07.4) |  |
| 5. How often do you feel that you are ignored or not taken seriously by your boss? | |  |  |  | 0.9606 |
| Never | 575 (64.8) | 445 (64.5) | 94 (65.7) | 36 (66.7) |  |
| Less than once a year | 99 (11.2) | 79 (11.4) | 13 (09.1) | 07 (13.0) |  |
| A few times a year | 103 (11.6) | 82 (11.9) | 15 (10.5) | 06 (11.1) |  |
| A few times a month | 48 (05.4) | 37 (05.4) | 08 (05.6) | 03 (05.6) |  |
| At least once a week | 20 (02.2) | 16 (02.3) | 03 (02.1) | 01 (01.8) |  |
| Almost every day | 42 (04.7) | 31 (04.5) | 10 (07.0) | 01 (01.8) |  |
| 6. How often have you been unfairly humiliated in front of others at work? |  |  |  |  | 0.2840 |
| Never | 702 (79.0) | 547 (79.0) | 110 (76.9) | 45 (83.3) |  |
| Less than once a year | 103 (11.6) | 85 (12.3) | 13 (09.1) | 05 (09.3) |  |
| A few times a year | 45 (05.1) | 30 (04.3) | 11 (07.7) | 04 (07.4) |  |
| A few times a month | 21 (02.4) | 16 (02.3) | 05 (03.5) | 00 (0) |  |
| At least once a week | 11 (01.2) | 10 (01.4) | 01 (0.7) | 00 (0) |  |
| Almost every day | 07 (0.8) | 04 (0.6) | 03 (02.1) | 00 (0) |  |
| Note: Baseline stress measures combined the HRS waves 2006 and 2008 since the PLB questionnaires were randomly administered to half the sample in each wave. | | | | |  |

| **Supplementary Table 4: Distribution of Life course items at baseline by race** | | | | | |
| --- | --- | --- | --- | --- | --- |
|  | **All Races (N=3904)** | **White (N=3159)** | **Black (A/A) (N=574)** | **Other (N=171)** |  |
| **Life course stress questions** | **N (%)** | **N (%)** | **N (%)** | **N (%)** | **p-value** |
| 1.Has a child of yours ever died? |  |  |  |  | <0.0001 |
| No | 3013 (78.3) | 2523 (80.8) | 364 (65.2) | 126 (74.6) |  |
| Yes | 836 (21.7) | 599 (19.2) | 194 (34.8) | 43 (25.4) |  |
| 2.Have you ever been in a major fire, flood, earthquake or other natural disaster |  |  |  |  | 0.7987 |
| No | 3064 (79.5) | 2478 (79.3) | 450 (80.5) | 136 (80.0) |  |
| Yes | 790 (20.5) | 647 (20.7) | 109 (19.5) | 34 (20.0) |  |
| 3.Have you ever fired a weapon in combat or been fired upon in combat? |  |  |  |  | 0.0096 |
| No | 3488 (90.3) | 2807 (89.6) | 523 (93.6) | 158 (92.4) |  |
| Yes | 374 (09.7) | 325 (10.4) | 36 (06.4) | 13 (07.6) |  |
| 4.Has your spouse, partner, or child ever been addicted to drugs or alcohol? |  |  |  |  | 0.0235 |
| No | 3078 (80.3) | 2516 (81.0) | 440 (78.6) | 122 (73.0) |  |
| Yes | 756 (19.7) | 591 (19.0) | 120 (21.4) | 45 (27.0) |  |
| 5.Were you the victim of a serious physical attack or assault in your life? |  |  |  |  | 0.0002 |
| No | 3588 (93.0) | 2929 (93.7) | 512 (91.6) | 147 (86.0) |  |
| Yes | 268 (07.0) | 197 (06.3) | 47 (08.4) | 24 (14.0) |  |
| 6.Did you ever have a life-threatening illness or accident? |  |  |  |  | < 0.0001 |
| No | 2364 (61.7) | 1864 (60.0) | 391 (70.1) | 109 (63.7) |  |
| Yes | 1469 (38.3) | 1240 (40.0) | 167 (29.9) | 62 (36.3) |  |
| 7.Did your spouse or a child of yours ever have a life- threatening illness or accident? |  |  |  |  | 0.0215 |
| No | 2570 (67.4) | 2055 (66.4) | 395 (71.6) | 120 (72.3) |  |
| Yes | 1245 (32.6) | 1042 (33.6) | 157 (28.4) | 46 (27.7) |  |
| 8.Before you were 18 years old, did you have to do a year of school over again? |  |  |  |  | 0.2285 |
| No | 3243 (83.7) | 2610 (83.2) | 488 (85.8) | 145 (85.8) |  |
| Yes | 633 (16.3) | 528 (16.8) | 81 (14.2) | 24 (14.2) |  |
| 9.Before you were 18 years old, did either of your parents drink or use drugs so often that it caused problems in the family? |  |  |  |  | 0.0495 |
| No | 3315 (85.7) | 2673 (85.2) | 503 (88.9) | 139 (83.2) |  |
| Yes | 554 (14.3) | 463 (14.8) | 63 (11.1) | 28 (16.8) |  |
| 10.Before you were 18 years old, were you ever physically abused by either of your parents? |  |  |  |  | 0.2914 |
| No | 3627 (93.6) | 2929 (93.3) | 540 (95.0) | 158 (94.0) |  |
| Yes | 247 (06.4) | 209 (06.7) | 28 (05.0) | 10 (06.0) |  |
| 11.Before you were 18 years old, were you ever in trouble with the police? * |  |  |  |  | 0.2752 |
| No | 1816 (95.4) | 1480 (95.6) | 256 (95.9) | 80 (92.0) |  |
| Yes | 87 (04.6) | 69 (04.4) | 11 (04.1) | 07 (08.0) |  |
| Notes: Baseline stress measures combined the HRS waves 2006 and 2008 since the PLB questionnaires were randomly administered to half the sample in each wave. *= Question asked beginning in HRS 2008; score was rescaled for HRS 2006 to make it easy to compare data between 2006 and 2008. | | | | | |

| **Supplementary Table 5: Distribution of recent stress items at baseline by race** | | | | | |
| --- | --- | --- | --- | --- | --- |
|  | **All Races (N=3904)** | **White (N=3159)** | **Black (A/A) (N=574)** | **Other (N=171)** |  |
| **Recent stress question** | **N (%)** | **N (%)** | **N (%)** | **N (%)** | **p-value** |
| 1.Have you involuntarily lost a job for reasons other than retirement at any point in the past five years? |  |  |  |  | 0.7511 |
| No | 3724 (95.8) | 3013 (95.7) | 549 (96.3) | 162 (95.3) |  |
| Yes | 165 (04.2) | 136 (04.3) | 21 (03.7) | 08 (04.7) |  |
| 2.Have you been unemployed and looking for work for longer than 3 months at some point in the past five years? |  |  |  |  | 0.2393 |
| No | 3746 (96.3) | 3041 (96.6) | 543 (95.3) | 162 (95.3) |  |
| Yes | 143 (03.7) | 108 (03.4) | 27 (04.7) | 08 (04.7) |  |
| 3.Was anyone else in your household unemployed and looking for work for longer than 3 months in the past 5 years? |  |  |  |  | < 0.0001 |
| No | 3638 (93.7) | 2980 (94.8) | 515 (90.5) | 143 (84.6) |  |
| Yes | 244 (06.3) | 164 (05.2) | 54 (09.5) | 26 (15.4) |  |
| 4.Have you moved to a worse residence or neighborhood in the past five years? |  |  |  |  | 0.8781 |
| No | 3816 (98.0) | 3091 (98.0) | 558 (97.7) | 167 (97.7) |  |
| Yes | 80 (02.0) | 62 (02.0) | 13 (02.3) | 04 (02.3) |  |
| 5.Were you robbed, or did you have your home burglarized in the past five years? |  |  |  |  | 0.0002 |
| No | 3709 (95.4) | 3024 (96.0) | 531 (93.3) | 154 (90.6) |  |
| Yes | 180 (04.6) | 126 (04.0) | 38 (06.7) | 16 (09.4) |  |
| 6.Have you been the victim of fraud in the past five years?* |  |  |  |  | 0.3362 |
| No | 1811 (95.2) | 1478 (95.4) | 252 (94.7) | 81 (92.0) |  |
| Yes | 92 (04.8) | 71 (04.6) | 14 (05.3) | 07 (08.0) |  |
| Notes: Baseline stress measures combined the HRS waves 2006 and 2008 since the PLB questionnaires were randomly administered to half the sample in each wave. *= Question asked beginning in HRS 2008; score was rescaled for HRS 2006 to make it easy to compare data between 2006 and 2008. | | | | | |

## Mediation analysis

| **Supplementary Table 6: Mediation analysis on impact of toxic stressors association between race and SRH declines.** | | | | | | |
| --- | --- | --- | --- | --- | --- | --- |
|  | **Toxic stressor** |  | **OR (95% CI)** | **p-Value Stressor** | **p-Value T*Stressor** | **Proportion due to TS** |
| **Race** | **None** | Black/AA vs White/Caucasian | **1.459 (1.252, 1.702)** | < 0.0001 | 0.6575 |  |
|  |  | Other vs White/Caucasian | **1.429 (1.095, 1.864)** |  |  |  |
|  |  | Black/AA vs Other | 1.021 (0.762, 1.370) |  |  |  |
|  |  |  |  |  |  |  |
| **Race** | **Life course stressors** | Black/AA vs White/Caucasian | **1.464 (1.252, 1.710)** | < 0.0001 | 0.8762 | 0% |
|  |  | Other vs White/Caucasian | **1.432 (1.093, 1.877)** |  |  | 0% |
|  |  | Black/AA vs Other | 1.022 (0.758, 1.378) |  |  | 0% |
|  |  |  |  |  |  |  |
| **Race** | **Recent stressors** | Black/AA vs White/Caucasian | **1.456 (1.249, 1.698)** | 0.487 | 0.3133 | 0% |
|  |  | Other vs White/Caucasian | **1.422 (1.089, 1.857)** |  |  | 0% |
|  |  | Black/AA vs Other | 1.024 (0.763, 1.375) |  |  | 0% |
|  |  |  |  |  |  |  |
| **Race** | **Life time discrimination** | Black/AA vs White/Caucasian | **1.377 (1.179, 1.609)** | < 0.0001 | 0.1087 | 6% |
|  |  | Other vs White/Caucasian | **1.379 (1.055, 1.804)** |  |  | 3% |
|  |  | Black/AA vs Other | 0.998 (0.742, 1.342) |  |  | 2% |
|  |  |  |  |  |  |  |
| **Race** | **Day to day discrimination** | Black/AA vs White/Caucasian | 1.463 (1.253, 1.707) | 0.0052 | 0.7197 | 0% |
|  |  | Other vs White/Caucasian | 1.450 (1.107, 1.899) |  |  | -1% |
|  |  | Black/AA vs Other | 1.009 (0.749, 1.359) |  |  | 1% |
| Note: Proportion due to TS for effect of Race= ((OR_Race_-OR_TS_)/ OR_Race_) *100 | | | | | | |
